# A New Remedy in Diphtheria

**Published:** 1880-12

**Authors:** 


### A NEW REMEDY IN DIPHTHERIA.

Dr. George Guttmann, writes in *Berlin. Klin. Wochenschrift*, that knowledge of the physiological action of pilocarpin and of its effect upon bronchial catarrh, giving rise to moist rales, led him to believe that administered in diphtheria, it might loosen the diphtheritic membrane through the induced abundant salivary secretion while it would not excite any inflammatory condition. The result of the proposed treatment was above all expectation, brilliant and striking.

April, 1879, an entire family of seven fell sick, one after another, with diphtheria; three exhibited the severest typhoid symptoms. The second case I treated with pilocarpin in moderate doses. The next day I found a copious salivation, and fragments of the pseudo-membrane floated in the expectorated matters. Pilocarpin was administered also to the other five patients. In addition, the usual general treatment was followed: quinine, tannin locally, gargles of lime water and pepsin. The patients recovered in from two to four days.

Since these first cases, down to August, 1880, sixty-six cases of diphtheria (rachenbräune) have come under my care; fifteen exhibited the worst of the diphtheritic symptoms, of which, according to my previous experience, at least two-thirds would have certainly died; thirty-three would be termed bad cases, the membrane being extensive; the remainder were slightly affected. I gave pilocarpin to all, only in the first cases, quinine and gargles also, they recovered as a rule, in periods of time varying from twenty-four hours to three days; of the fifteen worst cases, two recovered in nine and eleven days, the rest in two to five days. All patients who came early under treatment while the pseudo-membrane was still loosely adherent, without exception, were cured in within twenty-four hours. The doubt that these cases were not truly diphtheritic is not to be raised, since they were examined with the utmost care, and in the worst cases the contagion could be distinctly traced. Under the action of the pilocarpin, not only were the membrane and infiltration dissolved in the salivary flow, but also the violent inflammatory condition yielded to its influence, the deeply reddened, dry, mucous membrane soon became moist, pale red and in every respect of normal appearance.

Led by these results, I prescribed pilocarpin in violent pharyngeal cases, angina aphthosa and tonsillaris, always with most happy results, the disease yielding in a short time. In two cases of violent tonsillitis, in which the tonsils were so swollen that water could be taken only with great difficulty and scarification was positively indicated, not only did the swelling disappear, but the entire group of inflammatory symptoms, the one in twenty-four hours, and the other in thirty-six.

In the few cases of membranous croup that have fallen into my hands during the past fifteen months, pilocarpin has proved a faithful ally, and I believe it will prove as effective as in diphtheria of the fauces.

Two cases of laryngitis stridula yielded promptly to the same drug, which is safer and more convenient than the usually prescribed emetic.

Others have used pilocarpin under my advise, and agree with me in maintaining its excellence in diseases of the nature described. In the administration of this remedy I combine pepsin to combat the gastric catarrh usually present. My formula is as follows:

℞

| | | | |
|---|---|---|---|
| 1. | Pilocarpin muriat. | grams. | 0.02—0.04. |
| 2. | Pepsin. | " | 0.6 —08. |
| 3. | Acidi hydrochlor., | gtt. | ii. |
| 4. | Aquæ dest. | grams. | 80.0. |

M. Sig. A teaspoonful hourly for children.

For adults.

| | | |
|---|---|---|
| Pilocarpin muriat. | | 0.03—0.05. |
| Pepsin, | | 2.0. |
| Acidi hydrochlor., | gtt. | iii. |
| Aquæ dest. | | 240.0. |

S. Hourly, a tablespoonful.

I have never observed any undesirable effects of the drug even when it has been continued until complete recovery, possibly because I give a small amount of generous wine after each dose.—*St. Louis Courier of Medicine.*

